# Plasma diamine oxidase level predicts 6-month readmission for patients with hepatitis B virus-related decompensated cirrhosis

**DOI:** 10.1186/s12985-019-1219-4

**Published:** 2019-09-18

**Authors:** Feng-Cai Li, Yu-Chen Fan, Yue-Kai Li, Kai Wang

**Affiliations:** 1 Department of Hepatology, Qilu Hospital of Shandong University, Wenhuaxi Road 107#, Jinan, 250012 China; 2 Institute of Hepatology, Shandong University, Wenhuaxi Road 107#, Jinan, 250012 China; 3 Department of Nuclear Medicine, Qilu Hospital of Shandong University, Wenhuaxi Road 107#, Jinan, 250012 China

**Keywords:** Decompensated liver cirrhosis, Hepatitis B virus, Intestinal microecology, Diamine oxidase, Readmission

## Abstract

**Background and aims:**

Hepatitis B virus-related decompensated cirrhosis is difficult to cure but has a high readmission rate due to multiple complications. Our aim was to investigate the diagnostic potential value of plasma diamine oxidase (DAO) for 6-month readmission of patients with HBV-related decompensated cirrhosis.

**Methods:**

A total of 135 patients with HBV-related decompensated cirrhosis were prospectively collected at the onset of discharge of hospital, and then were followed up for at least 6 months with the readmission as the primary outcome. The plasma DAO level was measured using enzyme linked immunosorbent assay. In addition, 120 age and sex matched patients with HBV-related compensated cirrhosis were included as controls.

**Results:**

A total of 36 patients (36.7%) with decompensated cirrhosis admitted to hospital during the 6-month follow up. The plasma DAO level of readmission group [21.1 (14.5; 29.0) ng/ml] was significantly higher than that in the non-readmission group [12.7 (9.3; 18.0) ng/mL, *P* < 0.001]. Multivariate analysis showed that the plasma DAO level (HR = 1.102, *P* < 0.05) and hepatic encephalopathy (HE) (HR = 5.018, *P* < 0.05) were independent factors for 6-month readmission of decompensated cirrhosis. DAO level showed higher area under the curve of receiver operating characteristic (AUROC) than HE (0.769 vs. 0.598, *P* < 0.05) and Child-Pugh-Turcotte (CPT) score (0.769 vs. 0.652, *P* < 0.05) for predicting 6-month readmission rate, with the best cut-off value as 19.7 ng/mL. Furthermore, plasma DAO level (HR = 1.184, *P* < 0.05) was an independent factor and has the higher AUROC than CPT score for the onset of recurrent HE (0.905 vs. 0.738, *P* < 0.05) during the 6-month follow up.

**Conclusions:**

Plasma DAO level > 19.7 ng/mL predicts high rate of 6-month readmission in patients with HBV-related decompensated cirrhosis.

## Introduction

Liver cirrhosis is a chronic, progressive, diffuse fibrosis of the liver which is mainly caused by hepatitis B virus (HBV) infection in China [1, 2]. Annually, 3% ~ 5% of liver cirrhosis will develop to the decompensated state from compensated state, and the 5-year survival rate of decompensated cirrhosis is only about 14% ~ 35% [3]. Patients with decompensated cirrhosis would experience the frequent hospitalization and eventually die of ascites, gastrointestinal bleeding, spontaneous peritonitis (SBP), hepatic encephalopathy (HE), etc. [4]. Liver cirrhosis causes huge economic burden to society and family, decompensated cirrhosis causes more economic burden because of repeated hospitalization [5]. Therefore, it is very important to assess the liver function of liver cirrhosis patients in detail and clarify the factors of readmission. Currently, Child-Pugh-Turcotte (CPT) score system is mainly used to evaluate the liver function of liver cirrhosis patients, which reflects the liver function based on ascites, encephalopathy, albumin, bilirubin, clotting function [6], among them, there are subjective factors for the classification of ascites and hepatic encephalopathy, which may affect the determination of CPT values. Further, the CPT score system is not based on the Chinese cohort, and cannot predict and evaluate the readmission rate of patients with HBV-related decompensated cirrhosis. Therefore, new indicators are urgently needed to assess the readmission to hospital and severity for HBV-related decompensated cirrhosis.

At present, many studies have suggested that the imbalance of intestinal microecology is involved in the occurrence and development of multiple complications of decompensated cirrhosis [7–11]. The liver and the intestine are closely connected through the “Gut-Liver axis” [12]. In cirrhosis the intestinal microenvironment is directly or indirectly affected by liver damage and portal hypertension. Studies have shown that the stability of intestinal micro-ecological system is mainly related to the following aspects: excessive growth of intestinal bacteria, increased permeability of intestinal wall and decreased immunity [13, 14]. Increased permeability of intestinal wall can cause intestinal bacteria or their metabolites to shift and enter the body’s blood circulation, entering the liver through the portal system, further impairing liver function, leading to complications such as SBP, HE and other complications [15].

DAO is a kind of protein with molecular weight of 84,000 [16]. DAO is a highly active intracellular enzyme, closely related to nucleic acid and protein synthesis in the intestinal mucosa. Luk GD et al. first confirmed that plasma DAO is an indicator of intestinal mucosal maturation and barrier function in rats in 1980 [17]. Subsequently, many scholars confirmed that the changes of DAO activity could reflect the damage and repair of intestinal mucosal barrier function [18, 19]. So far, plasma DAO level has been demonstrated to be related to the development, progression and prognosis of many diseases, such as lung small-cell carcinoma and inflammatory bowel disease [20, 21]. Our previous work demonstrated that plasma DAO level can predict the short-term survival rate of acute-on-chronic hepatits B liver failure (ACHBLF) [22]. At present, it has been suggested that plasma DAO level is related to liver cirrhosis [23, 24].

However, there is no data on plasma DAO as a biomarker for predicting readmission rate in HBV-related decompensated cirrhosis. In this study, plasma DAO levels in decompensated cirrhosis patients were measured to determine whether the plasma DAO was associated with the severity and complications of HBV-related decompensated cirrhosis. In addition, decompensated cirrhosis patients were followed up for at least 6 months to further evaluate the diagnostic value of DAO as a biological marker in the readmission rate of HBV-related decompensated cirrhosis within 6 months.

## Methods

### Research design and research characters

**Fig. 1:**
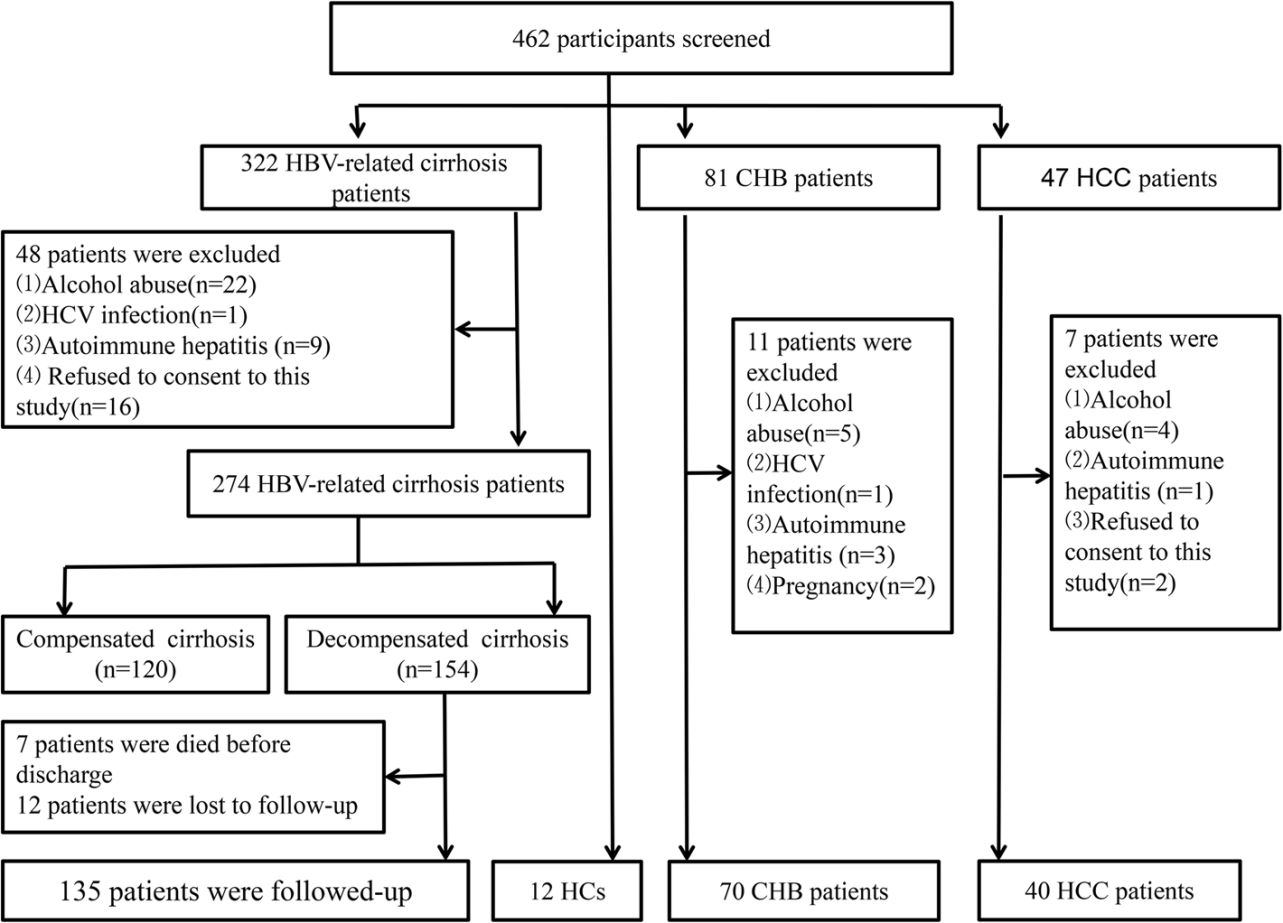
Flowchart depicting the selection and exclusion process of the participants

A total of 135 newly diagnosed HBV-related decompensated cirrhosis patients were prospectively enrolled at Department of Hepatology, Qilu Hospital of Shandong University from January 2014 to December 2016. In addition, 70 age and sex matched Chronic Hepatitis B (CHB) patients, 120 HBV-related compensated cirrhosis patients, 40 hepatocellular carcinoma (HCC) patients and 12 healthy controls were included as controls. According to the guidelines of the Helsinki declaration, all participants were approved in writing by the local research and ethics committee of Qilu hospital of Shandong University [25] The flow chart of selecting and excluding research objects was shown in Fig. 1.

According to evidence-based clinical practice guidelines for liver cirrhosis 2015, HBV-related decompensated cirrhosis diagnosed standard: (1) Serum hepatitis B surface antigen (HBsAg) positive ≥6 months; (2) liver histology or ultrasonography and other imaging suggested cirrhosis; (3) obvious decompensated liver function; (4) severe complications of cirrhosis, such as gastrointestinal bleeding, massive ascites, hepatorenal syndrome, SBP or HE. HBV-related compensated cirrhosis was diagnosed standard: (1) HBsAg positive ≥6 months; (2) liver histology or ultrasonography and other imaging suggested cirrhosis; (3) abnormal liver function or portal hypertension, including hypersplenism and varicose esophageal and gastric fundus; (4) no serious complications of cirrhosis such as esophageal varices, ascites or HE [26]. The exclusion criteria were as follows: (1) alcohol abuse; (2) confirmed to merge with other hepatotropic infections; (3) autoimmune liver disease; (4) reject participate in this study.

The start date of follow-up was the diagnosed date of HBV-related decompensated cirrhosis. All decompensated patients were followed up by telephone and the hospital case retrieval system for at least 6 months. The end point of follow-up was readmission to hospital or not. During the follow-up, 7 patients died before the discharge, 12 patients were lost follow-up because they could not be contacted after discharge.

### Measurement of plasma DAO level using enzyme-linked immunosorbent assay (ELISA)

On the morning of the second day of admission, 5 mL of fasting venous blood of study paricipants with EDTA as the anticoagulant was obtained. Centrifuged at 2000 r/min for 5 min at room temperatureand and stored in refrigerator at − 80 °C. The plasma DAO level was determined using the highly sensitive Human diamine oxidase (DAO) enzyme-linked immunosorbent assay (ELISA) kit (Lengton, shanghai, China) according to the manufacturer’s instructions. Briefly, 50uL standard, 40uL plasma samples were added into 96-well plated, respectively, 10uL anti-DAO antibody were added into the sample well, 50 uL Streptavidin-HRP were added into 96-well plated, respectively. The wells were then covered with the seal plate membrane, gently mixed and incubated at 37 °C for 60 min. The contents of the wells were then aspirated and rinsed with wash buffer repeatedly five times. 50 uL of detection reagent A was added to each well, and then detection reagent B 50 uL was added to each well, and incubated for 30 min at 37 °C. Finally, 50uL stop solution was added to each well to terminate the reaction. The absorbance (OD value) of each hole was measured sequentially at the wavelength of 450 nm within 10 min, and the DAO concentration was calculated. The sensitivity of the DAO ELISA kit is 0.78 ng/mL.

### Clinical characteristics

Alanine aminotransferase (ALT), aspartate aminotransferase (AST), total bilirubin (TBIL), albumin (ALB) and creatinine (Cr) were measured by COBAS integra 800 (Roche Diagnostics, Germany). PTA was quantifified using ACL TOP 700 (Instrument Laboratory, Lexington, USA). Hepatitis B surface antigen (HBsAg) and hepatitis B e antigen (HBeAg) were measured by COBAS 6000 analyzer series (Roche Diagnostics, Basel, Switzerland). HBV-DNA load was measured by ABI 7300 PCR System (Applied Biosystems, USA). WBCs, HGB, PLTs were measured by Sysmex XE-2100 (Chuoku, Kobe, Japan). All the clinical parameters were measured using standard laboratory methods at the Department of Laboratory Medicine, Qilu Hospital of Shandong University.

### Statistical analysis

The IBM SPSS19.0 software (SPSS Inc., Chicago, USA) and MedCalc 15.6 software (MedCalc Software, Ostend, Belgium) were used for statistical analysis. The Kolmogorov-Smirnov test was used to determine whether the data came from a normally distributed population. Categorical variables were represented by frequencies (%). Continuous variables were represented by median (centile 25; centile 75). Chi-square test was used for Categorical variables, and Mann–Whitney U test and the Wilcoxon signed rank test were used for continuous variables. Spearman correlation analysis was used to analyze the correlation between DAO and clinical and experimental data. Univariate Cox proportional hazards regression analyses was used to determine the risk factors for readmission of HBV-related decompensated cirrhosis patients, and covariates with *P* < 0.05 were incorporated into the multivariate Cox proportional hazards regression analyses to determine the independent risk factors for readmission of HBV-related decompensated cirrhosis patients. Receiver operating characteristic (ROC) curve was used to evaluate the diagnostic value of DAO in the readmission of HBV-related decompensated cirrhosis patients. The readmission time for the cut-off values was compared using the Kaplan-Meier method. Bilateral *P* < 0.05 was considered statistically significant.

## Results

### Basic parameters of the reasearch population

This study includes 135 patients with HBV-related decompensated cirrhosis. Meanwhile, 120 HBV-related compensated cirrhosis patients, 70 CHB patients, 40 HCC patients and 12 HCs as controls. The basic parameters of the research object were shown in Table 1.

**Table 1:** Characteristics of the research objects

	LC (*n* = 255)		CHB (*n* = 70)	HCC (*n* = 40)	HCs (*n* = 12)	*P* value
	Decompensated (*n* = 135)	Compensated (*n* = 120)				
Male* (%)	96 (71.1%)	84 (70%)	51 (72.9%)	32 (80%)	9 (75%)	0.145
Age (year)	63 (53.5–68)	58 (50–62)	49 (40–54)	55.5 (39.25–66)	50 (40.25–51.5)	0.217
HBeAg* (+), n(%)	90 (66.7%)	63 (52.5%)	49 (70%)	20 (50%)	NA	0.152
HBsAg	4416 (3325–7081)	4453 (3566–6266)	5746 (2926–7081)	4478.5 (3785–62,00)	NA	0.081
HBV-DNA* (+), n(%)	99 (73.3%)	90 (75%)	59 (84.3%)	32 (80%)	NA	0.095
ALT (IU/L)	78.5 (63–129.5)	111.5 (72.25–237.75)	282 (238–468)	66.5 (39.5–238.25)	31 (24–34.5)	0.000
AST (IU/L)	70.5 (51–96)	86.5 (51.25–113.5)	210.5 (183–359)	53 (36.25–133.75)	33 (25.25–37.75)	0.015
TBIL (μmol/L)	39.1 (26.2–58.7)	36.85 (23.85–49.45)	20.9 (14.6–27.5)	37 (19–73.25)	13.25 (12.525–15.225)	0.003
ALB (g/L)	31.35 (29.32–34.95)	34.75 (32.4–36.9)	44.6 (42.4–51)	36.9 (31–43.63)	47.6 (43.525–51.775)	0.000
PTA (%)	67 (60–84)	81.5 (74–90.75)	92 (89.5–104)	79.5 (64.5–90)	91 (85.75–101.25)	0.000
Cr (μmol/L)	75 (66.5–89.75)	74 (66.5–86.5)	76.5 (70–87)	88 (70.25–92.25)	70.5 (64.25–79.75)	0.216
WBC (10^9^/L)	4.82 (3.16–9.34)	5.87 (4.48–7.29)	6.64 (5.28–7.53)	4.57 (3.315–5.65)	6.85 (5.0–7.66)	0.383
HGB (g/L)	119.5 (107–135.75)	130 (117–140.75)	148 (145–151)	130 (108–136)	146.5 (135.75–149.75)	0.000
PLT (10^9^/L)	90.5 (55–105)	101 (88.25–144.5)	168.5 (145–205)	124 (76–200)	197.5 (176.5–239.5)	0.000
SBP*, (n,%)	21 (15.6%)	NA	NA	NA	NA	NA
Ascites*, (n,%)	112 (83%)	NA	NA	NA	NA	NA
GI bleeding*, (n,%)	27 (20%)	NA	NA	NA	NA	NA
HE*, (n,%)	18 (13.3%)	NA	NA	NA	NA	NA
Child-pugh A/B/C*, n(%)	0/90/45 (0/66.7%/33.3%)	72/48/0 (60%/40%/0%)	NA	NA	NA	0.000

*HBeAg* Hepatitis B e antigen, *HBsAg* Hepatitis B surface antigen, *ALT* Alanine aminotransferase, *AST* Aspartate aminotransferase, *TBIL* Total bilirubin, *ALB* Albumin, *PTA* Prothrombin activity; *Cr* Creatinine, *WBC* White blood cell, *HGB* Haemoglobin, *PLT* Platelet, *SBP* Spontaneous bacterial peritonitis, *GI* bleeding, Gastrointestinal bleeding, *HE* Hepatic encephalopathy

Quantitative variables were expressed as the median (centile 25; centile 75)

* Categorical variables were expressed as n (%)

The 6-month readmission rate for patients with HBV-related decompensated cirrhosis was 26.7%(36/135). We compared the clinical indicators between readmission group and non-readmission group, as shown in Table 2. We discovered that the readmission group had lower median levels of ALB [30.9 (26.7–34.6) g/L vs. 34.6 (32.0–37.9) g/L, *P* < 0.05] and HGB [115.5 (89.5–129.8) g/L vs. 125.0 (111.0–137.0) g/L, *P* < 0.05] than non-readmission group. Meanwhile, in readmission group, the incidences of SBP (27.8% vs. 11.1%, *P* < 0.05), GI bleeding (41.7% vs. 12.1%, *P* < 0.001) and HE (27.8% vs. 8.1%, *P* < 0.05) were higher than non-readmission group. The rate of Child-pugh socre C level was higher in readmission group than non-readmission (55.6% vs. 25.3, *P* < 0.05). There were no significant difference in sex, age, HBeAg, HBsAg, HBV-DNA, ALT, AST, TBIL, PTA, Cr, WBCs and PLT between readmission group and non-readmission group.

**Table 2:** Characteristics of the HBV-related decompensated cirrhosis patients

	Readmission (*n* = 36)	Non-Readmission (*n* = 135)	*P* Value
Male* (%)	28(77.8%)	68(68.7%)	0.303
Age (year)	59(53–61.6)	58(53–61)	0.574
HBeAg(+)*, n(%)	27(75%)	63(63.6%)	0.215
HBsAg	4122.5(3276.3–6266.0)	4477.0(3634.0–6326.0)	0.282
HBV-DNA(+)*, n(%)	27(75%)	72(72.7%)	0.792
ALT (IU/L)	72.0(54.3–109.5)	85.0(65.0–132.0)	0.105
AST (IU/L)	91.0(55.0–145.5)	79.0(45.0–114.0)	0.307
TBIL (μmol/L)	41.4(26.1–67.9)	39.8(27.0–56.9)	0.508
ALB (g/L)	30.9(26.7–34.6)	34.6(32.0–37.9)	0.042
PTA (%)	70.0(61.3–85.8)	75.0(63.0–88.0)	0.233
Cr (μmol/L)	89.0(68.5–93.8)	73.0(66.0–87.0)	0.087
WBC (10^9^/L)	4.5(2.9–9.0)	4.7(3.0–11.4)	0.807
HGB (g/L)	115.5(89.5–129.8)	125.0(111.0–137.0)	0.019
PLT (10^9^/L)	73.5(66.0–123.3)	90.0(54.0–133.0)	0.464
SBP*, (n,%)	10(27.8%)	11(11.1%)	0.018
Ascites*, (n,%)	31(86.1%)	81(81.8%)	0.557
GI bleeding*, (n,%)	15(41.7%)	12(12.1%)	0.000
HE*, (n,%)	10(27.8%)	8(8.1%)	0.003
Child-pugh A/B/C*, n(%)	0/16/20(0%/44.4%/55.6%)	0/74/25(0/74.7%/25.3%)	0.001

Quantitative variables were expressed as the median (centile 25; centile 75)

* Categorical variables were expressed as n (%)

### Plasma DAO level of study populations

**Fig. 2:**
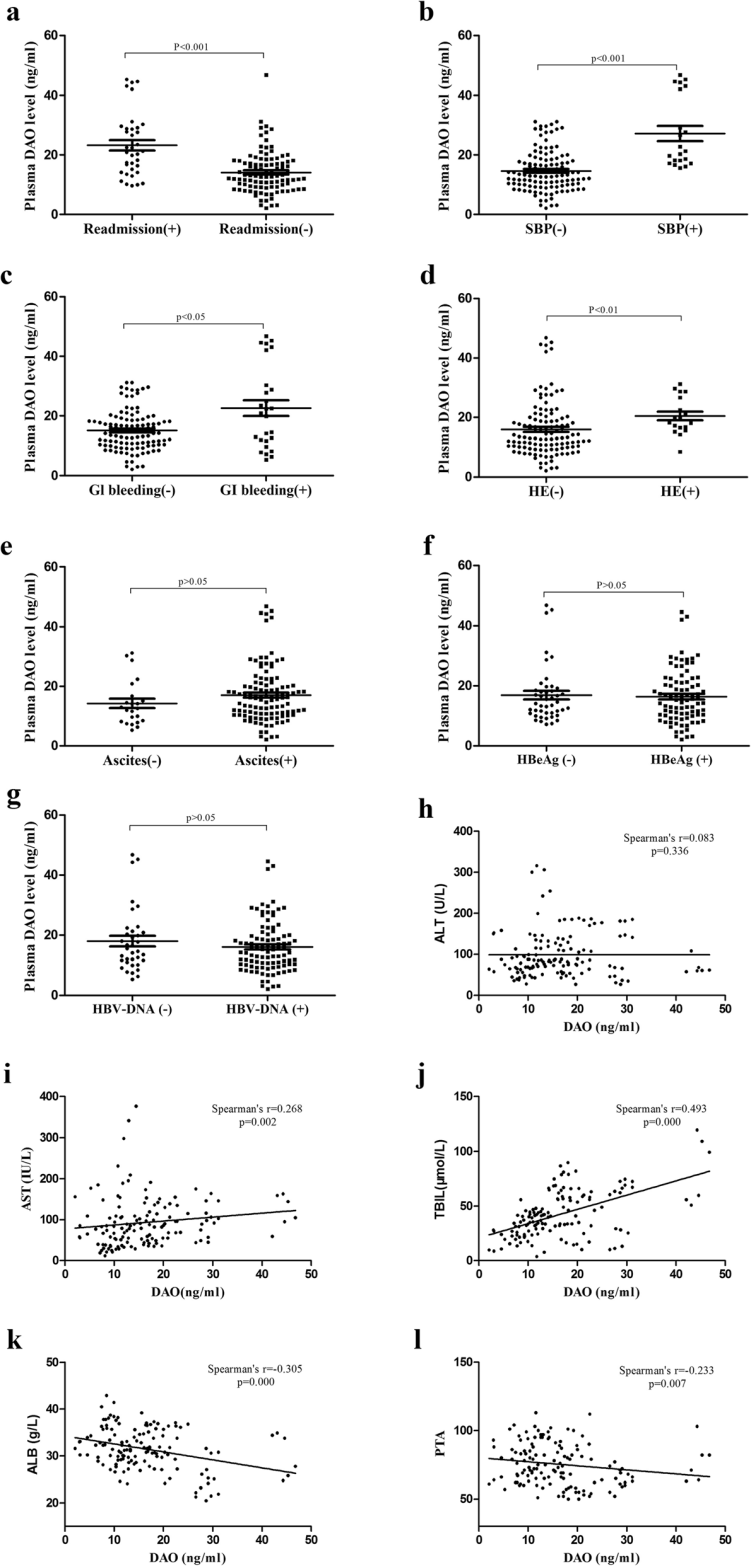
The relationship between DAO level and clinical parameters in HBV-related decompensated cirrhosis. **a,** The plasma DAO level of readmission group with 6 month was higher than non-readmission group (*P* < 0.001). **b**, The plasma DAO level of SBP group was higher than non-SBP group (P < 0.001). **c,** The plasma DAO level of GI bleeding group was higher than non-GI bleeding group (*P* < 0.05). **d**, The plasma DAO level of HE group was higher than non-HE group (*P* < 0.01). **e**, There was no significant difference in DAO level between ascites group and non-ascites group (*p* > 0.05). **f**, There was no significant difference in DAO level between HBeAg possitive group and negative group (*p* > 0.05). **g**, There was no significant difference in DAO level between HbeAg possitive group and negative group (*p* > 0.05). **h**, There was no significant associations between DAO and ALT (*p* > 0.05). **i**, The DAO level was positively correlated with AST (*r* = 0.268, *p* = 0.002). **j**, The DAO level was positively correlated with TBIL (*r* = 0.493, *p* = 0.000). **k,** The DAO level was negatively correlated with ALB (*r* = − 0.305, p = 0.000). **l**, The DAO level was negatively correlated with PTA (*r* = − 0.233, *p* = 0.007)

We detected the plasma DAO level of all study population. The DAO level of readmission group [21.1 (14.5; 29.0) ng/mL] was higher than non-readmission group [12.7 (9.3; 18.0) ng/mL, *P* < 0.001] (Fig. 2a). The plasma DAO level of patients with decompensated cirrhosis [14.6 (10.4; 20.3) ng/mL] was significantly higher than patients with compensated cirrhosis [9.4 (6.2; 13.5) ng/mL, *P* < 0.001]. The plasma DAO level of patients with liver cirrhosis [11.8 (7.9; 17.7) ng/mL] was significantly higher than CHB patients [4.9 (3.3; 6.9) ng/mL, *P* < 0.001], and lower than HCC patients [18.3 (13.4; 20.5) ng/mL, *P* < 0.001]. The plasma DAO level of liver cirrhosis patients with child-pugh B [12.6 (9.3; 16.4) ng/mL] was higher than child-pugh A [7.8 (5.4; 9.9) ng/mL, *P* < 0.001], and lower than child-pugh C [22.4 (16.5; 29.1) ng/mL, *P* < 0.001].

### The realtionship between plasma DAO level and clinical parametes in HBV-related decompensated cirrhosis

The DAO level of readmission group was higher than non-readmission group [21.1 (14.5; 29.0) vs. 12.7 (9.3; 18.0) ng/mL, *P* < 0.001], as shown in Fig. 2a. The plasma DAO levels in SBP group, GI bleeding group, and HE group were higher than those non-SBP group[21.3 (17.6; 42.6) vs. 13.0 (9.7; 18.5) ng/mL, *P* < 0.001], non-GI bleeding group [20.9 (11.6; 30.3) vs. 14.2 (10.3.; 18.6) ng/mL, *P* < 0.05] and non-HE group [19.0 (16.3; 27.1) vs. 13.3 (10.0; 19.5) ng/mL, *P* < 0.05], respectively. However, Plasma DAO levels were no statistically difference in the ascites-positive group, HBeAg-positive group, HBV-DNA positive group and ascites-negative group [16.0 (10.8; 20.3) vs. 12.6 (8.2; 16.7) ng/mL, *P* > 0.05], HBeAg-negative group [15.2 (10.1; 21.3) vs. 14.1 (10.9; 18.9) ng/mL, *P* > 0.05], and HBV-DNA negative group [15.5 (10.2; 19.7) vs. 15.1 (11.2; 21.2) ng/mL, *P* > 0.05], respectively, as shown in Fig. 2b-g. DAO level was positively correlated with AST (*r* = 0.268, *p* = 0.002) and TBIL (*r* = 0.493, *p* = 0.000), negatively correlated with ALB (*r* = − 0.305, *p* = 0.000) and PTA(*r* = − 0.233, *p* = 0.007). However, there was no significant associations between DAO level and ALT (*r* = 0.083, *p* = 0.336) (Fig. 2h-l).

### The plasma DAO level was an inependent risk factor for readmission within 6 months of HBV-related decompensated cirrhosis

The 6-month readmission rate for patients with HBV-related decompensated cirrhosis was 26.7%(36/135). Among the readmission patients, 5 had HE, 3 had HE with ascites, 1 had HE and GI bleeding, 12 had GI bleeding, 3 had GI bleeding and ascites, 2 had ascites,10 had ascites and SBP, as shown in the Table 3.

**Table 3:** The characters of the readmission patients with hepatitis B virus-related decompensated cirrhosis

Cases	Gender	Age(years)	Child-pugh score(A/B/C)	SBP(+/−)	Ascites(+/−)	GI bleeding(+/−)	HE(+/−)	Readmission Reasons	Readmission Time (months)
1	Male	55	C	+	+	+	–	HE and ascites	0.25
2	Male	53	B	–	+	+	–	GI bleeding	4.75
3	Male	41	C	–	+	–	+	HE	0.95
4	Female	61	C	–	+	+	+	SBP and ascites	5.40
5	Male	55	B	–	–	+	–	GI bleeding	5.20
6	Male	64	C	+	+	+	–	HE	2.80
7	Male	53	C	+	+	–	–	GI bleeding and HE	3.10
8	Male	61	B	+	+	–	–	HE and ascites	2.30
9	Male	57	B	–	+	–	–	HE and ascites	4.45
10	Male	55	C	+	+	+	–	SBP and ascites	1.75
11	Male	55	B	–	+	–	–	GI bleeding	1.35
12	Female	61	C	–	+	+	+	SBP and ascites	1.40
13	Female	61	C	–	+	+	+	GI bleeding	1.70
14	Male	62	B	–	+	–	+	GI bleeding	3.05
15	Male	62	B	–	+	–	+	SBP and ascites	3.05
16	Male	64	C	+	+	+	–	HE	5.65
17	Male	64	C	+	+	+	–	GI bleeding and ascites	1.15
18	Male	53	C	+	+	–	–	ascites	2.10
19	Female	66	C	–	+	+	–	SBP and ascites	1.45
20	Female	66	C	–	+	+	–	GI bleeding	1.05
21	Male	53	C	+	+	–	–	SBP and ascites	5.10
22	Male	41	C	–	+	–	+	HE	0.95
23	Male	53	B	–	+	+	–	GI bleeding and ascites	2.35
24	Male	61	B	+	+	–	–	GI bleeding	4.30
25	Female	66	C	–	–	+	–	SBP and ascites	1.05
26	Male	57	B	–	+	–	–	GI bleeding	4.45
27	Male	53	B	–	+	–	–	ascites	4.25
28	Male	61	B	–	–	–	+	SBP and ascites	3.95
29	Male	61	B	–	–	–	+	GI bleeding	3.65
30	Male	41	C	–	+	–	+	HE	1.53
31	Male	58	C	–	–	+	–	GI bleeding	2.50
32	Female	39	B	–	+	–	–	GI bleeding	3.05
33	Male	59	C	–	+	–	–	GI bleeding	2.95
34	Female	59	B	–	+	–	–	SBP and ascites	4.25
35	Male	59	C	–	+	–	–	GI bleeding	2.95
36	Male	69	B	–	+	–	–	SBP and ascites	5.10

We used univariate Cox regression analyses to detemine the risk factors for 6-month readmission of HBV-realted decompented cirrhosis, as shown in Table 4. The plasma DAO level (HR = 1.126, *P* < 0.001), the Child-pugh score (HR = 3.700, *P* < 0.05), GI bleeding (HR = 5.179, *P* < 0.001), HE (HR = 4.375, *P* < 0.001), SBP (HR = 3.077, *P* < 0.001) and HGB (HR = 0.979, *P* < 0.05) were the risk factors for the 6-month readmission of HBV-related decompensated cirrhosis. Then the above risk factors were analyzed by Cox Forward regression analyses, and it was found that the DAO level (HR = 1.102, *P* < 0.05) and HE (HR = 6.018, *P* < 0.05) were independent risk factors for readmission within 6 months of HBV-realted decompensated cirrhosis.

**Table 4:** Uni- and multivariate Cox analysis of factors associated with readmission in HBV-related decompensated cirrhosis patients

variable	univariate			mutlivariate		
	HR	95%CI	*P*	HR	95%CI	*P*
Gender (male/female) *	0.627	0.257–1.531	0.305			
Age (year)	1.066	0.957–1.057	0.814			
HBeAg(+/−)*	1.000	0.445–2.246	1.000			
HBsAg	1.000	1.000–1.000	0.669			
HBV-DNA(+/−)*	1.125	0.469–2.697	0.792			
ALT(IU/L)	0.994	0.986–1.002	0.120			
AST(IU/L)	1.001	0.995–1.007	0.763			
TBIL(umol/L)	1.010	0.993–1.028	0.246			
PTA	0.984	0.959–1.010	0.223			
ALB(g/L)	0.959	0.880–1.044	0.334			
WBC(10^9^/L)	1.053	0.952–1.165	0.313			
HGB(g/L)	0.979	0.963–0.995	0.010	0.980	0.942–1.019	0.313
PLT(10^9^/L)	0.996	0.987–1.004	0.302			
SBP(+/−)*	3.077	1.176–8.049	0.022	2.230	0.561–8.870	0.255
Ascites(+/−)*	0.494	0.192–1.286	0.143			
HE(+/−)*	4.375	1.567–12.215	0.000	5.018	1.436–17.533	0.012
GI Bleeding(+/−)*	5.179	2.113–12.690	0.000	1.520	0.156–14.788	0.718
Chilid-pugh score(B/C) *	3.700	1.665–8.223	0.001	0.914	0.281–2.972	0.881
DAO(ng/mL)	1.126	1.068–1.188	0.000	1.102	1.023–1.188	0.010

*HR* Hazard ratio, *CI* Confidence interval.

* Categorical variables

To further determine the relationship between the plasma DAO level and the causes of readmission within 6 months, Cox regression analysis was performed for readmission of different causes. In HBV-related decompensated cirrhosis, plasma DAO (HR = 1.184, *P* < 0.05) was an independent risk factor for readmission due to HE within 6 months, as shown in Table 5. Plasma DAO level was associated with readmission due to ascites within 6 months, but was not an independent risk factor, as shown in Table 6. Plasma DAO level was not associated with readmission due to GI bleeding within 6 months, as shown in Table 7. Plasma DAO was associated with 6-month readmission due to SBP, but was not an independent risk factor, as shown in Table 8.

**Table 5:** Uni- and multivariate Cox analysis of factors associated with readmission due to HE in HBV-related decompensated cirrhosis patients

variable	univariate			mutlivariate		
	HR	95%CI	*P*	HR	95%CI	*P*
Gender (male/female) *	0.000	0.000	0.998			
Age (year)	0.940	0.869–1.017	0.125			
HBeAg(+/−)*	0.603	0.154–2.365	0.468			
HBsAg	1.000	1.000–1.000	0.762			
HBV-DNA(+/−)*	0.426	0.108–1.682	0.223			
ALT(IU/L)	1.002	0.991–1.013	0.673			
AST(IU/L)	1.006	0.997–1.015	0.179			
TBIL(umol/L)	1.028	1.000–1.057	0.049	0.985	0.950–1.020	0.391
PTA	0.949	0.897–1.004	0.068			
ALB(g/L)	0.974	0.838–1.132	0.733			
WBC(10^9^/L)	1.115	0.948–1.312	0.188			
HGB(g/L)	0.992	0.965–1.019	0.550			
PLT(10^9^/L)	0.994	0.978–1.010	0.450			
SBP(+/−)*	8.594	2.087–35.394	0.003	1.138	0.133–9.732	0.906
Ascites(+/−)*	1.692	0.201–14.228	0.628			
HE(+/−)*	3.700	0.836–16.37	0.085			
GI Bleeding(+/−)*	2.125	0.596–9.109	0.310			
Chilid-pugh score(B/C) *	8.105	1.609–40.828	0.011	1.886	0.081–9.733	0.921
DAO(ng/mL)	1.157	1.078–1.243	0.000	1.184	1.024–1.369	0.023

*HR* Hazard ratio, *CI* Confidence interval

* Categorical variables

**Table 6:** Uni- and multivariate Cox analysis of factors associated with readmission due to ascites in HBV-related decompensated cirrhosis patients

variable	univariate			mutlivariate		
	HR	95%CI	*P*	HR	95%CI	*P*
Gender (male/female) *	0.939	0.311–2.837	0.911			
Age (year)	1.053	0.979–1.132	0.164			
HBeAg(+/−)*	1.351	0.450–4.057	0.592			
HBsAg	1.000	1.000–1.000	0.565			
HBV-DNA(+/−)*	1.318	0.404–4.302	0.648			
ALT(IU/L)	0.989	0.976–1.002	0.088			
AST(IU/L)	1.002	0.994–1.010	0.595			
TBIL(umol/L)	1.008	0.986–1.030	0.467			
PTA	0.995	0.962–1.028	0.746			
ALB(g/L)	0.941	0.842–1.052	0.284			
WBC(10^9^/L)	1.071	0.944–1.215	0.286			
HGB(g/L)	0.988	0.969–1.009	0.256			
PLT(10^9^/L)	0.997	0.986–1.008	0.559			
SBP(+/−)*	3.400	1.109–10.419	0.032	1.309	0.286–5.989	0.729
Ascites(+/−)*	0.473	0.150–1.489	0.201			
HE(+/−)*	2.102	0.606–7.290	0.242			
GI Bleeding(+/−)*	4.126	1.442–11.810	0.008	2.359	0.685–8.126	0.174
Chilid-pugh score(B/C) *	2.929	1.066–8.044	0.037	1.002	0.255–3.931	0.998
DAO(ng/mL)	1.090	1.036–1.146	0.001	1.065	0.987–1.150	0.105

*HR* Hazard ratio, *CI* Confidence interval.

* Categorical variables

**Table 7:** Uni- and multivariate Cox analysis of factors associated with readmission due to GI Bleeding in HBV-related decompensated cirrhosis patients

variable	univariate			mutlivariate		
	HR	95%CI	*P*	HR	95%CI	*P*
Gender (male/female) *	0.532	0.143–1.982	0.347			
Age (year)	1.006	0.940–1.077	0.863			
HBeAg(+/−)*	1.577	0.478–5.199	0.454			
HBsAg	1.000	1.000–1.000	0.787			
HBV-DNA(+/−)*	2.800	0.604–12.983	0.188			
ALT(IU/L)	0.998	0.989–1.008	0.750			
AST(IU/L)	0.999	0.990–1.008	0.835			
TBIL(umol/L)	0.995	0.971–1.020	0.711			
PTA	0.986	0.951–1.022	0.434			
ALB(g/L)	0.983	0.875–1.104	0.769			
WBC(10^9^/L)	1.039	0.907–1.190	0.584			
HGB(g/L)	0.972	0.951–0.993	0.008	0.974	0.935–1.014	0.200
PLT(10^9^/L)	0.994	0.982–1.006	0.334			
SBP(+/−)*	1.295	0.335–5.004	0.708			
Ascites(+/−)*	0.875	0.228–3.357	0.846			
HE(+/−)*	1.600	0.408–6.278	0.500			
GI Bleeding(+/−)*	3.850	1.284–11.548	0.016	1.122	0.131–9.578	0.916
Chilid-pugh score(B/C) *	1.658	0.574–4.786	0.350			
DAO(ng/mL)	1.032	0.980–1.086	0.235			

*HR* Hazard ratio, *CI* Confidence interval.

* Categorical variables

**Table 8:** Uni- and multivariate Cox analysis of factors associated with readmission due to SBP in HBV-related decompensated cirrhosis patients

variable	univariate			mutlivariate		
	HR	95%CI	*P*	HR	95%CI	*P*
Gender (male/female) *	2.676	0.729–9.827	0.138			
Age (year)	1.104	0.993–1.28	0.068			
HBeAg(+/−)*	0.732	0.196–2.738	0.643			
HBsAg	1.000	1.000–1.000	0.676			
HBV-DNA(+/−)*	0.837	0.204–3.427	0.805			
ALT(IU/L)	0.958	0.928–0.990	0.070			
AST(IU/L)	0.998	0.987–1.010	0.777			
TBIL(umol/L)	0.999	0.970–1.029	0.933			
PTA	1.007	0.966–1.049	0.755			
ALB(g/L)	0.926	0.802–1.069	0.294			
WBC(10^9^/L)	1.030	0.870–1.218	0.735			
HGB(g/L)	0.987	0.962–1.013	0.326			
PLT(10^9^/L)	1.002	0.989–1.016	0.748			
SBP(+/−)*	1.395	0.275–7.080	0.688			
Ascites(+/−)*	5.944	1.562–22.620	0.009	5.200	0.730–37.047	0.100
HE(+/−)*	5.286	1.327–21.050	0.018	3.529	0.616–20.224	0.157
GI Bleeding(+/−)*	4.682	1.248–17.566	0.022	1.704	0.239–12.169	0.595
Chilid-pugh score(B/C) *	3.308	0.883–12.389	0.076			
DAO(ng/mL)	1.064	1.004–1.127	0.037	1.072	0.990–1.160	0.085

*HR* Hazard ratio, *CI* Confidence interval

* Categorical variables

### The value of plasma DAO as a biomarker in the readmisson of HBV-related decompensated cirrhosis patients within 6-months

**Fig. 3:**
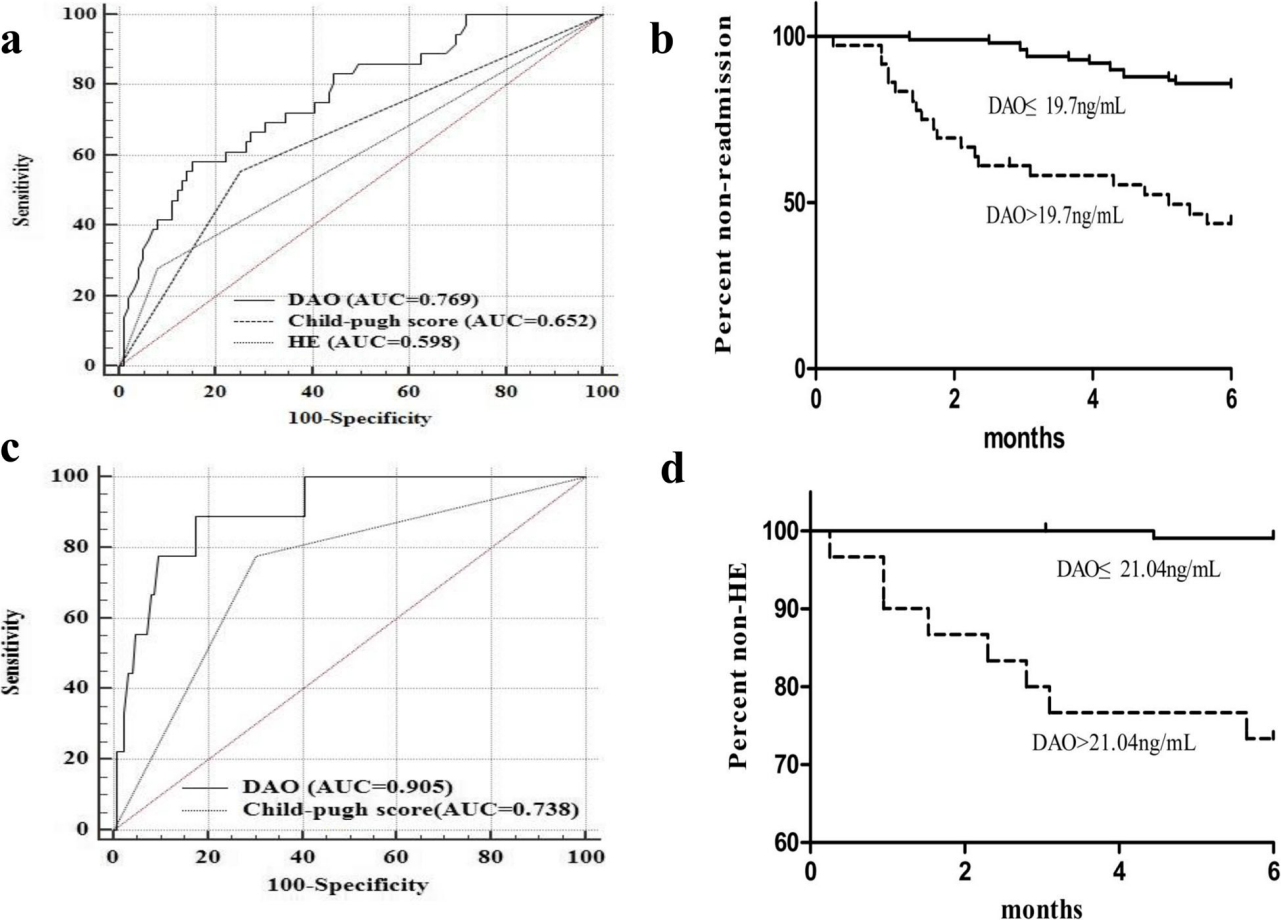
Sensitivities and specificities for plasma DAO for predicting 6-month readmission of HBV-related decompensated cirrhosis patients (**a**). Kaplan-Meier readmission curves for plasma DAO at the optimal cut-off value 19.7 ng/mL at the end of 6-month follow-up (**b**). Sensitivities and specificities for plasma DAO for predicting 6-month readmission due to HE of HBV-related decompensated cirrhosis patients (**c**). Kaplan-Meier recurrent HE curves for plasma DAO at the optimal cut-off value 21.04 ng/mL at the end of 6-month follow-up (**d**)

The readmission rate of HBV-related decompensated cirrhosis within 6 months was 26.7% (36/135). The mean readmission time of HBV-related decompensated cirrhosis at the end of 6-month was 2.924 (SE 0.255, 95% CI: 2.425–3.424) months. The AUROC for DAO was 0.769 (SE 0.0457, 95%CI: 0.689–0.837), which was significantly higher than HE [0.598 (SE 0.0403, 95%CI: 0.511–0.682, *P* < 0.05] and Child-Pugh score [0.652 (SE 0.0474, 95%CI: 0.565–0.731, *P* < 0.05], as shown in Fig. 3a. The plasma DAO level of 19.7 ng/mL, with a sensitivity of 58.33% and specificity of 84.35%, was selected as the cut-off value for readmission within 6 months in patients with HBV-related decompensated cirrhosis. Moreover, the readmission rate within 6 months of HBV-related decompensated cirrhosis patients with plasma DAO > 19.7 ng/mL group [58.3% (21/36)] was higher than plasma DAO ≤ 19.7 ng/mL group [15.2% (15/99)]. Futhermore, the readmission time with the level of plasma DAO > 19.7 ng/mL [1.75 months (SE 0.435, 95%CI: 0.898–2.602)] was statistically different from patients with plasma DAO ≤ 19.7 ng/mL [3.65 months (SE 0.386, 95%CI: 2.893–4.407); *P* < 0.05], as shown in Fig. 3b.

After 6-month follow-up, 6 of the 36 readmission HBV-related decompensated cirrhosis patients were readmitted due to HE. The DAO level of recurrent HE group was higher than non-recurrent HE group [29.7 (24.5; 43.4) vs. 14.1(10.1; 19.0) ng/mL, *P* < 0.001]. The mean recurrent HE time was 2.442 (SE 0.589, 95% CI: 1.084–3.801) months. The AUROC for DAO was 0.905 (SE 0.0452, 95%CI: 0.842–0.949), which was significantly higher than Child-Pugh score [0.738 (SE 0.0763, 95%CI: 0.655–0.810, *P* < 0.05], as shown in Fig. 3c. The plasma DAO level of 21.04 ng/mL, with a sensitivity of 88.9% and specificity of 82.5%, was selected as the cut-off value for recurrent HE within 6 months in patients with HBV-related decompensated cirrhosis. Moreover, the recurrent HE rate within 6 months with plasma DAO > 21.04 ng/mL group [23.3% (7/30)] was higher than plasma DAO ≤ 21.04 ng/mL group [1.9% (2/105), *P* < 0.05]. Futhermore, the readmission time with the level of plasma DAO > 21.04 ng/mL [2.176 months (SE 0.698, 95%CI: 0.469–3.883)] was statistically different from patients with plasma DAO ≤ 21.04 ng/mL [3.375 months (SE 1.075, 95%CI: 0.282–17.034); *P* < 0.05], as shown in Fig. 3d.

## Discussion

HBV-related decompensated cirrhosis is often accompanied by gastrointestinal bleeding, hepatic encephalopathy, spontaneous peritonitis and other complications, resulting in repeated hospitalization [27]. Thus causes the serious economic burden to the society and the family. At present, many models and markers have been used for the prognosis of decompensated cirrhosis and the diagnosis of readmission [28]. In recent years, Many researchers have proved that gut flora disorder exists in liver cirrhosis patients [11]. In addition, intestinal microecology is related to the complications of liver cirrhosis, such as SBP, HE and prognosis of liver cirrhosis [29, 30]. Plasma DAO is the most sensitive indicator to predict intestinal barrier function [19]. Our previous study confirmed that plasma DAO could be used as a biological indicator to predict the 1-month mortality of acute-on-chronic hepatitis B liver failure. However, there were few studies on the diagnostic value of plasma DAO level in the readmission of patients with HBV-related decompensated cirrhosis within 6 months. This study found for the first time that the plasma DAO level in HBV-related decompensated cirrhosis patients was significantly higher than that in compensated cirrhosis patients. After 6 months of follow-up, the plasma DAO level of the readmission patients was significantly higher than that of the non-readmission patients. We confirmed that plasma DAO level was an independent risk factor for 6-month readmission and predicted readmission for HBV-related decompensated cirrhosis with 6 months.

Imbalance of intestinal flora is involved in the occurrence and development of a variety of diseases [31]. The liver and intestinal have a close anatomical and functional relationship. Intestinal microbiota play an important role in the development of liver cirrhosis through the gut-liver axis and microbiota-liver axis [32, 33]. In patients with HBV-related cirrhosis, there are changes both in intestinal flora outcome and function [34]. Portal hypertension and intestinal congestion can lead to increased permeability of the intestinal wall [35]. When intestinal wall permeability increases, intestinal bacteria and their metabolites are more likely to enter extraintestinal organs, thus inducing complications such as HE and SBP [36]. Overgrowth of intestinal flora, bacteria translocation and increased permeability of intestinal wall promote each other, thus forming a vicious cycle [37]. Therefore, changes in intestinal wall permeability play an important role in HBV-related liver cirrhosis [15]. At present, the detection of intestinal wall permeability mostly used the indirect method. Plasma DAO was the most sensitive indicator to predict intestinal barrier function.

Our study found that plasma DAO levels were significantly elevated in patients with SBP, GI bleeding, and HE. Therefore, in patients with decompensated cirrhosis, plasma DAO levels reflect the severity of cirrhosis in patients. TLRs (Toll-like receptors) are natural immune receptors in the liver, mainly expressed in Kupffer cells, endothelial cells, dendritic cells, bile duct epithelial cells, hepatic stellate cells and hepatocytes [38]. There are many pathogen-associated molecular patterns (PAMPs) in the gut, and these PAMPs cannot reach the systemic circulation due to the presence of intestinal barrier function. Lipopolysaccharide (LPS) is a component of the cell wall of Gram-negative bacteria and is currently the most studied PAMPs. Studies have shown that when the intestinal barrier function is impaired, LPS enters the liver through the portal system, inducing activation of liver macrophage Kupffer cells. Kupffer cells trigger the release of factors such as TNF-α through NF-κB-mediated mechanisms which activates specific intracellular pathways through cascades, induce apoptosis, cause liver damage, and aggravate cirrhosis [39]. On the other hand, LPS, as a hepatic toxin, causes an acute inflammatory reaction in the liver, which is characterized by polymorphonuclear cell aggregation, neutrophils releasing reactive oxygen metabolites, proteases, etc., causing liver damage to be aggravated [40].

Our study found that in patients with HBV-related decompensated cirrhosis, plasma DAO levels were significantly higher in patients who were readmitted after 6 months of follow-up than those who did not. We used the Cox proportional hazards regression model to find that plasma DAO levels and HE were independent risk factors for readmission of patients with HBV-related decompensated cirrhosis within 6 months. Considering Child-pugh scores commonly used for liver function grading in patients with cirrhosis, we used K-M survival analysis to analyze the diagnostic value of plasma DAO levels, HE, and Child-pugh scores for readmission in patients with decompensated cirrhosis. We found that the AUROC of DAO was significantly higher than the HE and Child-Pugh scores. The reasons for readmission of patients who were readmitted to liver-related diseases within 6 months of follow-up were mainly HE, SBP, GI bleeding, and massive ascites. Analysis of the reasons, mainly have the following points: Liver cirrhosis and portal hypertension affect intestinal dysfunction, abnormal secretion of bile salts, leading to excessive growth of intestinal bacteria [33]; Intestinal flora structure of patients with cirrhosis and functional changes, such as the increase in the number of pathogenic Enterobacteriaceae and Streptococcus [9–11]; Increased intestinal permeability, leading to intestinal flora translocation [41]. Ammonia has long been regarded as a core factor in the pathogenesis of HE in patients with cirrhosis. The ammonia in the blood circulation is mainly derived from the decomposition of amino acids in the intestinal bacteria and the intestinal circulation of urea [42]. When the permeability of the intestinal wall increases, the amount of ammonia in the intestine increases, which increases the risk of HE. SBP is a dangerous complication of decompensated patients with cirrhosis [43]. Gram-negative bacteria are the most common pathogenic bacteria of SBP, accounting for about 80%, especially *Escherichia coli* [44], so it is believed that the infection of spontaneous bacterial peritonitis is mainly intestinal infection [45]. The translocation of bacteria to the mesenteric lymph nodes is an important starting point for SBP [13]. SBP is associated with impaired intestinal motility, intestinal bacterial overgrowth and intestinal barrier dysfunction. Intestinal barrier dysfunction is the most critical link. When intestinal barrier function is impaired, intestinal bacteria are increased. The possibility of translocation to the mesenteric lymph nodes, which has been confirmed in the rat liver cirrhosis model. In the mesenteric lymph nodes of a mouse model of cirrhosis, the researchers found bacteria homologous to spontaneous peritonitis [46].

To further validate the diagnostic value of plasma DAO for patients readmitted with HBV-related decompensated cirrhosis within 6 months, we took plasma DAO level of 19.7 ng/mL, sensitivity of 58.33%, specificity of 84.35%, as the cut-off value of patients with HBV-related decompensated cirrhosis within 6 months. We found that the readmission rate of DAO > 19.7 ng/mL patients was significantly higher than that of DAO ≤ 19.7 ng/mL group, and the cumulative readmission time of the two groups was statistically different. In summary, plasma DAO can predict readmission of patients with HBV-realted decompensated cirrhosis within 6 months.

However, our study had some limitations. The first and foremost, we did single-center research, not multi-center research. Second, this study without cohort validation. Third, the exact mechanism of DAO involvement in the process of liver cirrhosis was still unclear, and we will continue to conduct further research on this issue.

## Conclusions

In summary, we first demonstrated that the plasma DAO might be predict the readmission with 6 months of HBV-related decompengsated cirrhosis. Plasma DAO level > 19.7 ng/mL predicts high rate of 6-month readmission in patients with HBV-related decompensated cirrhosis.

## Data Availability

All relevant information is provided in this current manuscript.

## Abbreviations

ACHBLF: Acute-on-chronic hepatitis B liver failure
AFP: Alpha fetal protein
ALB: Albumin
ALT: Alanine aminotransferase
AST: Aspartate aminotransferase
AUC: The area under the receiver operating characteristic curve
CHB: Chronic hepatitis B
CPT: Child-Pugh-Turcotte
Cr: Creatinine
DAO: Diamine oxidase
ELISA: Enzyme-linked immunosorbent assay
GI bleeding: Gastrointestinal bleeding
HBeAg: Hepatitis B e antigen
HbsAg: Hepatitis B surface antigen
HBV: Hepatitis B virus
HC: Healthy control
HE: Hepatic encephalopathy
HGB: Haemoglobin
INR: International normalized ratio
MELD: Model for end-stage liver disease
PLT: Platelet
PTA: Prothrombin activity
ROC: Receiver operating characteristic curve
SBP: Spontaneous bacterial peritonitis
TBIL: Total bilirubin
WBC: White blood cell
